# Cellular Autofluorescence following Ionizing Radiation

**DOI:** 10.1371/journal.pone.0032062

**Published:** 2012-02-22

**Authors:** Dörthe Schaue, Josephine A. Ratikan, Keisuke S. Iwamoto

**Affiliations:** Radiation Oncology, David Geffen School of Medicine at University of California Los Angeles, Los Angeles, California, United States of America; University of Queensland, Australia

## Abstract

Cells often autofluoresce in response to UV radiation excitation and this can reflect critical aspects of cellular metabolism. Here we report that many different human and murine cell types respond to ionizing radiation with a striking rise in autofluorescence that is dependent on dose and time. There was a highly reproducible fluorescent shift at various wavelengths, which was mirrored by an equally reproducible rise in the vital intracellular metabolic co-factors FAD and NADH. It appears that mitochondria, metabolism and Ca^2+^ homeostasis are important for this to occur as cells without mitochondria or cells unable to alter calcium levels did not behave in this way. We believe these radiation-induced changes are of biological importance and that autofluorescence may even provide us with a tool to monitor radiation responses in the clinic.

## Introduction

Most cells and tissues are intrinsically fluorescent when exposed to UV radiation of certain wavelengths without the addition of external fluorophores. This autofluorescence has been largely localized to mitochondria and lysosomes as these organelles contain most of the endogenous fluorophores, in particular NAD(P)H and flavin coenzymes [1], [2]. While the reduced nicotinamide adenine dinucleotide(phosphate), NAD(P)H, fluoresces, it is the oxidized flavin adenine dinucleotide, FAD, that responds to excitation by ultraviolet light (UV). Any change in the equilibrium of these reduced/oxidized metabolites or their propensity to be free vs bound will shift a cell's autofluorescence signal [3]. Aromatic amino acids and lipo-pigments are additional sources of cellular autofluorescence and extracellular matrix components collagen and elastin contribute strongly to tissue autofluorescence. It follows that changes in almost any morphological and physiological state, be it oxidative stress, metabolism, cell growth and cell survival or death may alter autofluorescence [4], [5]. Indeed, diagnostic in vivo imaging techniques are already taking advantage of changes in tissue autofluorescence as a means to detect tumors [6]–[9].

Here we report that cells respond to ionizing radiation with striking and robust changes in autofluorescence. We believe this is important on more than one level: It may provide us with a new way to image radiation therapy response and even be a quantification tool in the context of accidental or incidental radiation exposures. It is also relevant to radiation experiments that use flow cytometry and fluorescent microscopy with externally added markers as significant changes in background staining may skew results and alter their interpretation. Above all, it may serve to monitor biologically relevant pathways through which a cell responds to radiation.

## Methods

### Ethics statement

Male or female 6- to 8- week old C57BL/6 gnotobiotic mice were bred and maintained in the American Association of Laboratory Animal Care-accredited facility of the Department of Radiation Oncology at the University of California, Los Angeles. All experimental protocols adhered to local and national animal care guidelines.

### Cell lines

The experiments described here are mostly based on the mouse dendritic cell line, DC2.4,that were a kind gift from Kenneth Rock [10] and cultured in RPMI (Mediatech, Herndon, VA) with 10% fetal bovine serum (FBS) (Omega Scientific, Tarzana, CA), 10,000IU penicillin, 10,000 µg/ml streptomycin, and 25 µg/ml amphotericin (Mediatech). The B16-OVA murine melanoma cell line was a kind gift from James Economou (UCLA) and the 3LL lewis lung carcinoma from Sherven Sharma (UCLA). Both lines were maintained in complete RPMI as above. MCaK murine breast carcinoma cells came from Kathy Mason, MD Anderson, Texas [11]. U87 human glioma, PC3 human prostate carcinoma, RAW264.7 murine macrophages and EG.7-OVA murine lymphoma cells were purchased from the American Type Culture Collection (ATCC, Manassas, VA) and were cultured in DMEM with 10% FBS, 10,000IU penicillin, 10,000 µg/ml streptomycin, and 25 µg/ml amphotericin. U87Rho(0) cells were generated from parental U87 cells by the depletion of mitochondrial DNA (mtDNA) via the well-established method of chronic incubation in the presence of ethidium bromide [12]. Briefly, U87 cells were grown in 50 ng/ml ethidium bromide for at least 6 months in complete DMEM medium supplemented with uridine (50 µg/ml) and sodium pyruvate (1 mM). Loss of mtDNA was confirmed by Southern blotting.

### Primary cells

Splenocytes from male or female 6- to 8- week old C57BL/6 mice were depleted of red blood cells by ammonium chloride treatment (ACK Lonza Walkersville Inc., MD) in serum-free RPMI-1640. Whole blood was taken from the posterior vena cava into heparinized tubes and peripheral blood mononuclear cells (PBMCs) were separated from red cells and granulocytes over a Ficoll™-based gradient (Atlanta Biologicals). Single cell suspensions of splenocytes, red cells and PBMCs in complete RPMI medium were used for experiments.

### Cell irradiation and flow cytometry

Cell were maintained on plastic dishes at subconfluent levels and irradiated at room temperature with a Gulmay X-ray unit at 300 kV and a beam filtration with 1.5 mm Cu and 3 mm Al filters (Gulmay Medical Ltd., Camberly, Surrey, UK) giving a HVL of 3 mm Cu at a dose rate of 2.879 Gy/min. Total doses of 0.1 Gy or less were given at a dose rate of 0.0568 Gy/min. Multiple dosimetric evaluations were performed using Harshaw TLD-100H (LiF:Mg, Cu, P) and film (GAFCHROMIC EBT2, International Specialty Products, Wayne, NJ) with NIST calibration against a clinical cobalt-60 irradiator (Theratron-1000, MDS Nordion, Ontario). The inhomogeneity of radiation dose within a field was less than 6.5%.

Prior to analysis, cells were harvested by mechanical dislocation and re-suspended in phosphate-buffered saline (Mediatech, Herndon, VA). Flow cytometric analysis was performed on 30,000 events using a FACSCalibur with a 488 nm laser (BD Biosciences, Mountain View, CA). Fluorescent signals in FL-1 were detected at 515–545 nm. Cell debris with very low forward/side scatter were excluded from the analysis. Where indicated, 7-Amino-Actinomycin D (7-AAD) was added 5–10 minutes prior to flow cytometry so as to exclude non-viable cells from the analysis.

### FAD and NAD/NADH assay

Intracellular FAD was measured using the FAD Assay and deproteinizing Sample Preparation Kit (BioVision Research Products, Mountain View, CA). Intracellular NAD and NADH were determined with the Fluoro NAD/NADH™ Detection Kit (Cell Technology, Mountain View, CA). Both kits were used according to the manufacturers instruction.

### Statistics

Data were analyzed for statistical significance using the two-tailed Student's t-test at the 5% level.

## Results

### Radiation raises cellular autofluorescence in a dose- and time dependent manner

**Figure 1:**
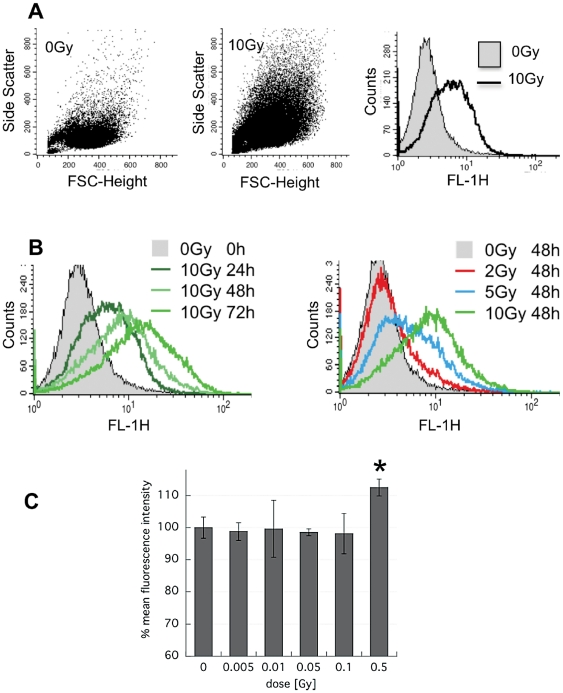
Radiation increases autofluorescence in a dose- and time- dependent fashion. DC2.4 cells were irradiated or not under standard culture conditions and analyzed by FACS. **A**) Forward-side scatter dot blot and Fl-1 histogram of untreated cells and cells at 24 h after treatment with 10 Gy. **B**) (left) Overlay histograms of FL-1 fluorescence in 10 Gy-irradiated cells analyzed at 24, 48 and 72 h and (right) overlay histogram of FL-1 fluorescence after 0, 2, 5 and 10 Gy and analyzed at 48 h. **C**) Mean FL-1 fluorescence intensity normalized to control of n = 2±s.d. in DC2.4 treated with radiation ranging from 0.005 to 0.5 Gy and analyzed at 48 h after exposure. *p<0.05.

Cells typically swell as a result of irradiation, which can easily be observed under the microscope or by flow cytometry. Our dendritic cell line DC2.4 was no exception. The forward- and side- scatter of cells had increased markedly by 24 h after exposure to 10 Gy, as did the FL-1 autofluorescence (Figure 1A). The increase in autofluorescence was remarkably reproducible and was further amplified when cells were incubated beyond 24 h (Figure 1B, left panel). There was a clear dose-dependency with doses below 10 Gy (Figure 1B, right panel). In fact, radiation doses as low as 0.5 Gy led to measurable autofluorescence augmentation (Figure 1C). Alterations detected in the FL-1 channel of the FACScalibur were mirrored by similar changes in FL-2(585 nm/42) and FL-3(650 nm) (not shown) as well as at multiple excitation/emission wavelengths using a 96-well plate reader (SpectraMax M5, Molecular Devices Corp., Sunnyvale, CA) (not shown).

### Changes in cellular autofluorescence are common

To determine if radiation-induced changes in autofluorescence were specific to DC2.4, a variety of other murine and human cell lines and murine primary hematopoietic cells were examined. All of the 13 cell types tested displayed this response with only one exception, namely erythrocytes. It is likely that fluorescence absorption <600 nm by the dense hemoglobin in erythrocytes may have obscured any such change in this cell type.

**Figure 2:**
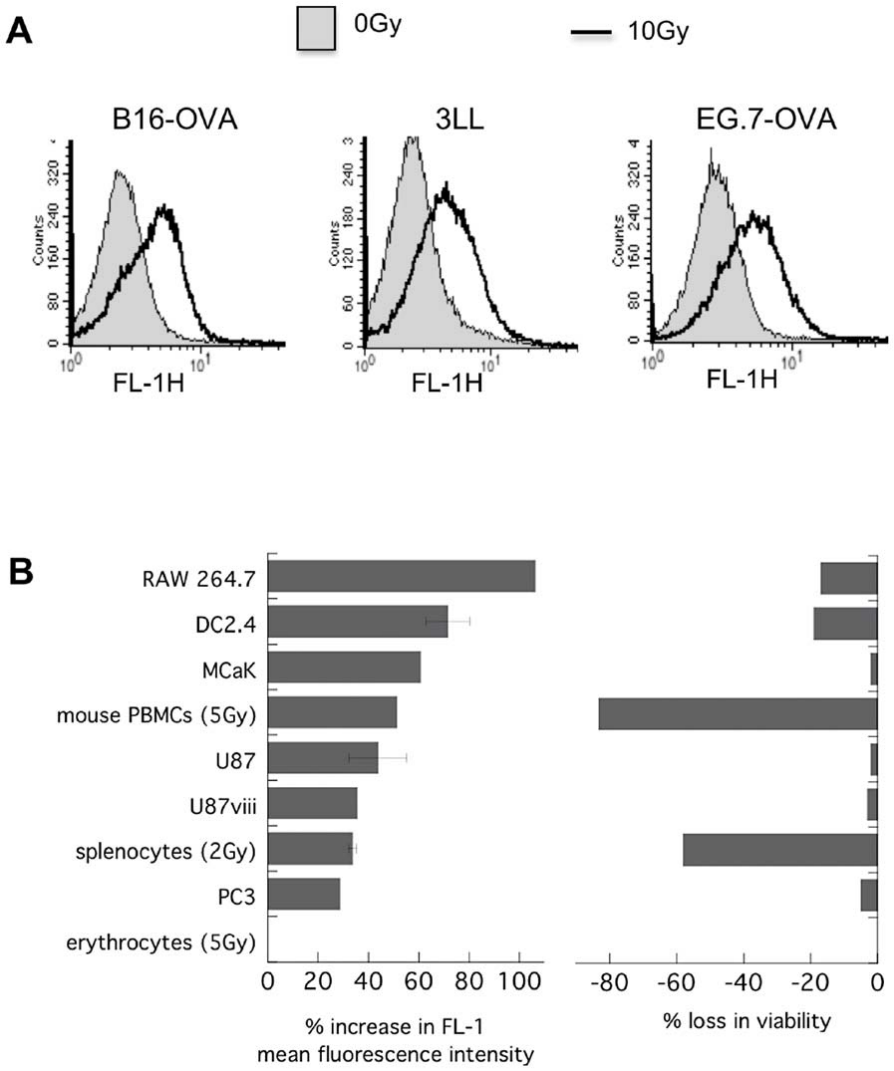
Many cell types respond to radiation with a rise in autofluorescence. Various murine and human cell lines and primary mouse peripheral blood cells were irradiated with 10 Gy (or less when indicated) and left under standard culture conditions for 24 h prior to FACS analysis. Autofluorescence was recorded in FL-1 and cell viability assessed according to 7-AAD dye exclusion in FL-3. **A**) FL-1 histogram overlays for treated and untreated B16-OVA, 3LL and EG.7-OVA cells. **B**) Percent increase in mean FL-1 fluorescence and percent loss in viability in the irradiated sample as compared to control of n = 1–8±s.e.m.

The extent of increased autofluorescence however varied between different cell types. Immune cells that have higher intrinsic radiation-sensitivity e.g. splenocytes and PBMCs tended to show a larger increase in autofluorescence while more radiation-resistant tumor cells such as U87 glioma or PC3 prostate cancer cells showed less autofluorescence responses (Figure 2B). Exclusion of dead cells from the analysis only marginally affected the magnitude of the autofluorescence increase (not shown).

### Irradiated cells have higher levels of FAD and NADH

**Figure 3:**
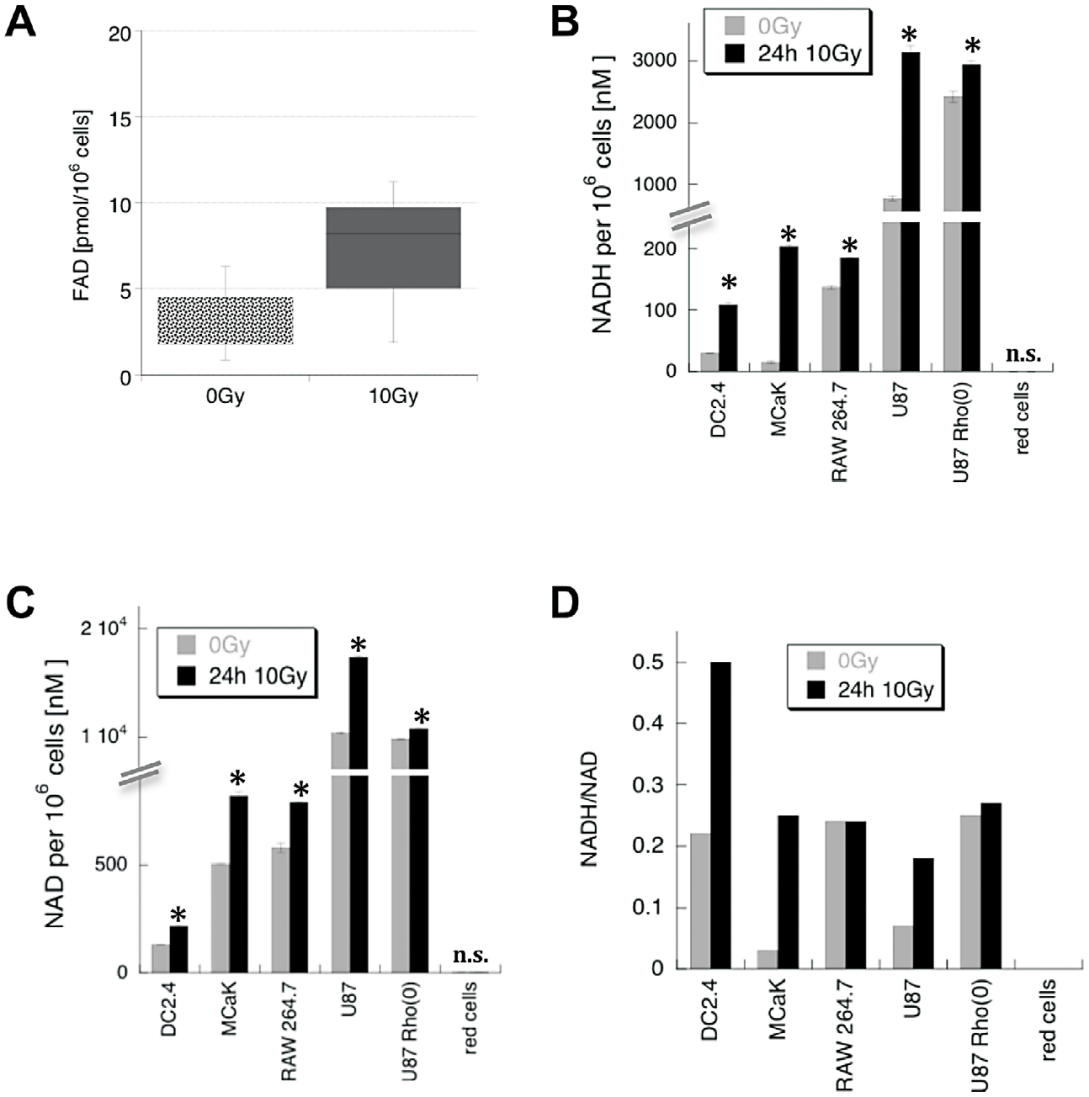
Intracellular FAD, NAD and NADH rise following irradiation. Cells were irradiated with 10 Gy or left untreated and incubated for 24 h before harvest and FAD or NAD/NADH assay. **A**) range of FAD concentrations per 1 million DC2.4. cells (n = 3), **B**) Mean NADH concentration per 1million cells (n = 2±s.d.), **C**) mean NAD concentration per 1 million cells of n = 2±s.d., **D**) ratio of mean NADH/NAD per 1 million cells (n = 2±s.d. *p<0.05).

Of all the known cell metabolites, the flavin nucleotide cofactor, FAD, is most likely to respond to 488 nm excitation with emission of green light [13]. Indeed, irradiated DC2.4 contained much higher FAD levels than control cells (Figure 3A). However, NADH is much more abundant than FAD and generally considered the major cellular source of autofluorescence, prompting us to scan a panel of cells lines for their levels of NADH and NAD. NADH and NAD content varied considerably between different cell lines with NAD always being much more abundant than NADH (Figure 3B and 3C). As expected, both, NADH and NAD were by far more prevalent than FAD, at least in DC2.4 cells. Importantly, the amount of NADH consistently went up on a per cell basis in all lines following irradiation with 10 Gy and in most cases, the radiation-induced increase was somewhat more pronounced for NADH than for NAD as evidenced by a rise of the NADH/NAD ratio (Figure 3D). Red cells were again the exception, having remarkably little observable baseline NADH/NAD and no measurable changes following irradiation. Taken together, absolute as well as relative amounts of NADH and FAD increased in irradiated cells mirroring the rise in autofluorescence.

### Mitochondria and calcium are important

**Figure 4:**
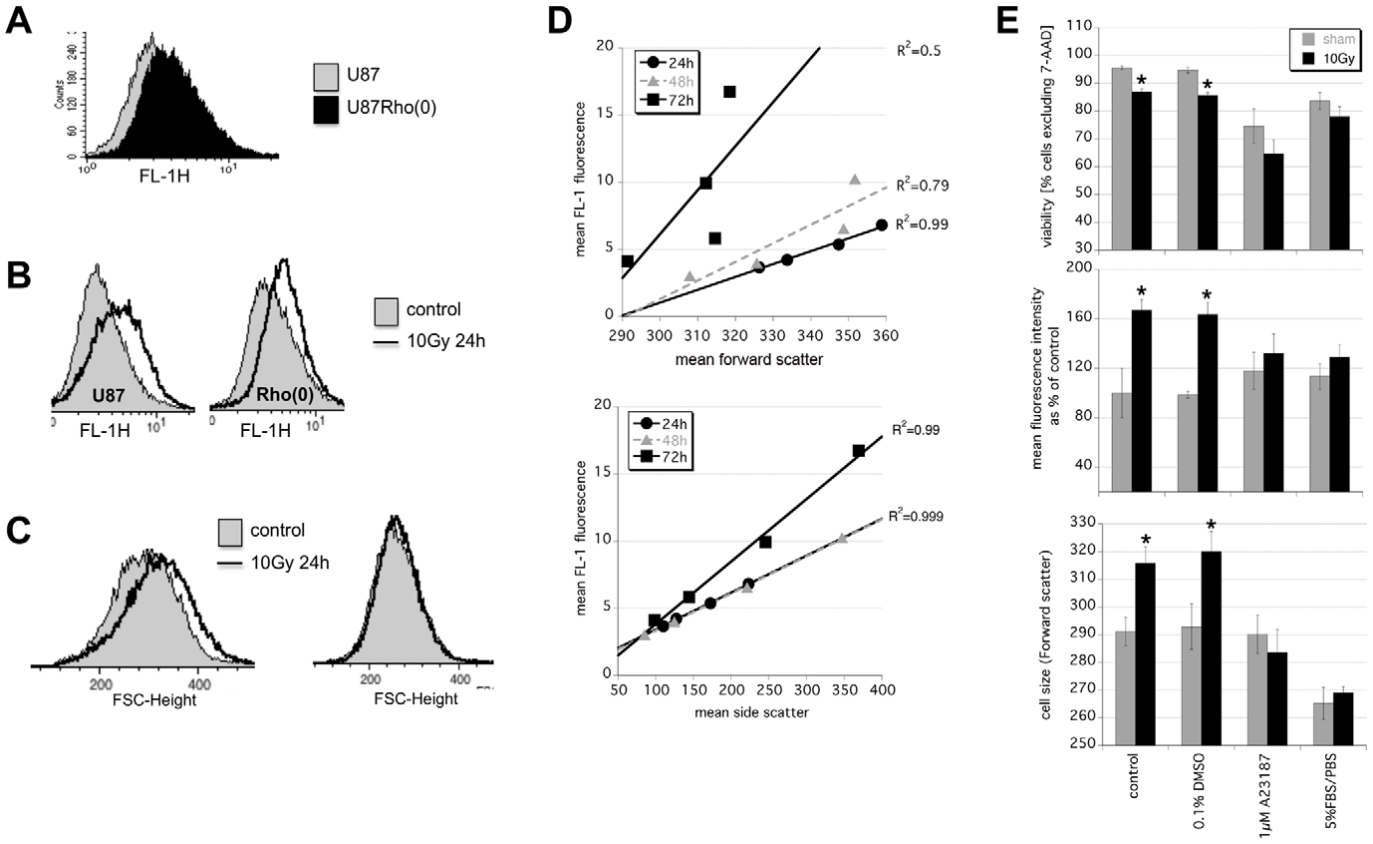
Mitochondria and calcium homeostasis are important for irradiated cells to change their autofluorescence. **A**) Overlay histogram comparing background FL-1 fluorescence in untreated U87 and U87Rho(0) cells. U87 and mitochondria-deficient U87Rho(0) were treated with 10 Gy and FACS-analyzed 24 h later for **B**) FL-1 autofluorescence and **C**) cell size. **D**) Radiation dose-dependent changes in FL-1 autofluorescence in irradiated DC2.4 after 0, 2, 5, and 10 Gy correlate with changes in forward scatter (top) and side scatter (bottom). **E**) DC2.4 cells were irradiated with 0 or 10 Gy in the presence of 1 µM A23187 or 0.1% DMSO or 5%FBS/PBS and analyzed 24 h later for viability by 7-AAD exclusion (top). Viable cells were compared for cell size (bottom) and FL-1 autofluorescence (middle). Data are mean ± s.e.m. of n = 6 *p<0.05.

FAD and NADH/NAD homeostasis are intricately linked with mitochondrial function, calcium flux, metabolism changes and cell death [14], [15]. We used U87 cells that lack mitochondria, U87Rho(0), to investigate the role of these organelles in the response to radiation. A direct comparison of parental U87 and U87Rho(0) cells showed that the latter had higher baseline NADH (Figure 3B) and higher baseline autofluorescence (Figure 4A). Both increased further in U87Rho(0) following 10 Gy irradiation, but not as much as in the parental line (Figure 3B and Figure 4B). The cellular swelling often seen following irradiation was also less obvious in the absence of mitochondria (Figure 4C).

It appears therefore that the dose-dependent rise in autofluorescence is tightly linked to changes in metabolism and cell morphology. The correlation between FL-1 autofluorescence and forward scatter of 0, 2, 5 and 10 Gy irradiated DC2.4 was strong at 24 h and 48 h (Figure 4D top, R^2^ = 0.99 and 0.97), as it was between FL-1 autofluorescence and side scatter (Figure 4D bottom, R^2^ = 0.99 and 0.99). Radiation-induced swelling can be prevented by keeping cells under low nutrient conditions (PBS/5% FBS) or by exposing them to the calcium ionophore A23187 (Figure 4E bottom); in both circumstances the radiation-induced rise in autofluorescence was also muted (Figure 4E middle). Clearly, mitochondrial changes in NAD/NADH, calcium homeostasis, cellular swelling, and autofluorescence are all part of an integrated cellular response to radiation.

## Discussion

Radiation is a cytotoxic agent and radiation-response pathways are classically defined by DNA damage and repair leading to cell survival or death. A lot of research aims to decipher the molecules and pathways that integrate this response as they ultimately decide the fate of a cell. Our observation that there is a rise in autofluorescence in irradiated cells, which is widespread amongst different cell types suggests a universal read-out for common underlying molecular response pathways.

The clear dose dependency for autofluorescence indicates that this response relates to cytotoxicity. This is supported by the fact that there was a threshold dose of 0.5 Gy below which an increase in autofluorescence couldn't be detected, suggesting a minimum level of damage may be necessary to drive this response. The exclusion of dead cells from the analysis did not significantly alter the conclusions and cell death per se is clearly not needed for the rise in autofluorescence but pathways leading to cell death may well be involved. Furthermore, there is a continuum within the increased autofluorescent profile indicating that all cells respond while only some die.

Perhaps the simplest explanation is that the rise in cellular autofluorescence following irradiation is linked to cell cycle arrest and a concomitant increase in size and protein accumulation, as is seen senescent cells [16], [17]. This is plausible also with respect to cellular changes in morphology and the apparent dose-dependent correlation between cell size, granularity and autofluorescence. It is also consistent with the observed rise in autofluorescence at many different wavelengths.

Cellular autofluorescence is a broad-based phenomenon with the biggest source being NADH [1]. However, NADH has an excitation/emission maximum of 340 nm/450 nm [3], [18] and is unlikely to be directly responsible for the FL-1 fluorescence fluctuations we observed with the blue/green 488 nm laser by FACS. The flavin nucleotide cofactor, FAD, on the other hand responds to excitation at 488 nm and emits green light when oxidized [13]. Given that oxidized FAD and reduced NADH are intimately linked in the respiratory chain, forming a tight redox equilibrium [19], we can assume that any change in fluorescence of one is analogous to changes in the other. This is speculative but reasonable because the radiation-driven rise in autofluorescence included the wavelength characteristic for NADH when measured on a multi-well plate reader (not shown) and because irradiated cells have much more FAD and NADH than before exposure.

NADH is central to a cell's metabolic status and one can hypothesize why NADH might change after irradiation: Radiation induces ATP release from cells [20] and the repair of DNA damage requires energy in the form of ATP as well as NAD building blocks for purposes such as poly-ADP-ribose polymerase (PARP) activation [21]. The resultant drop in ATP is likely to drive oxidative phosphorylation to provide more ATP and hence would use more NADH. While there is general consensus that direct radiation effects on the electron transport chain in vitro are unlikely [22]–[24], we frequently noted a temporary drop in NADH within the first few hours of radiation exposure along with a similar brief dip in autofluorescence (not shown). Interestingly, intrinsic fluorescence is also known to dip by 20% during the binding of the Ku protein to damaged DNA in the non-homologous end-joining DNA repair pathway [25].

Studies, primarily in the brain, have shown that a drop in NADH (and fluorescence) can in turn induce the tricarboxylic acid cycle followed by glycolysis with a NADH production replenishing overshoot [26]. It is therefore reasonable to assume that cells respond to radiation with an attempt to maintain their reducing power, be it through glycolysis (NADH) [27] or through the pentose phosphate pathway (NADPH) [28] and one could theoretically argue that such a compensatory restoration of reduction equivalents may eventually lead to this net increase in NAD(P)H and a later rise in fluorescence. Interestingly, NADH has the ability to inactivate PTEN and drive Akt, both of which are linked to cell swelling [29]–[31]. The net increase in reduction equivalents may also explain why the MTT assay is generally not optimal to assess radiation-induced cell death, as changes in the formation of the formazan dye might be masked by a rise in NAD(P)H levels [32].

Clearly, the proposed underlying mechanisms for the radiation-induced increase in cellular autofluorescence are highly speculative at this point, but it appears to be a biologically important phenomenon worthy of further investigation.
